# Identification and Evaluation of Non-Accidental Trauma in the Pediatric Population: A Clinical Review

**DOI:** 10.3390/children11040413

**Published:** 2024-03-30

**Authors:** Sophia M. V. Schermerhorn, Oliver J. Muensterer, Romeo C. Ignacio

**Affiliations:** 1 Naval Medical Center San Diego, San Diego, CA 92134, USA; sophia.m.schermerhorn.mil@health.mil; 2 LMU Medical Center, Pediatric Surgery, Dr. von Hauner Children’s Hospital, Ludwig-Maximilians-University, Lindwurmstrasse 4, 80337 Munich, Germany; oliver.muensterer@med.uni-muenchen.de; 3 Department of Surgery, UCSD School of Medicine, 9500 Gilman Dr, La Jolla, CA 92093, USA; 4 Division of Pediatric Surgery, Rady Children’s Hospital San Diego, 3020 Children’s Way, San Diego, CA 92123, USA

**Keywords:** non-accidental trauma, child abuse, child physical abuse, non-accidental injury, skeletal survey

## Abstract

Non-accidental trauma (NAT) is a major cause of morbidity and mortality for children around the world and most significantly impacts children under one year of age. Prompt and comprehensive treatment of these children relies on a high index of suspicion from any medical provider that treats pediatric patients. This review discusses those most at risk for experiencing NAT, and common initial presentations, to assist providers in the identification of potential victims. In addition, this review provides guidance on the recommended workup for these patients so that the full extent of associated injuries may be identified and the appropriate healthcare team may be assembled.

## 1. History and Definition

The formal recognition of the term “battered child” by the American Academy of Pediatrics in 1961 marked a pivotal moment in the medical community’s acknowledgment of a complex spectrum of pediatric injuries stemming from physical abuse [1]. Today, the comprehensive concept of “child abuse” is defined as “any action or omission by the adult caregiver or older adolescent that might result in damage to the child’s physical, emotional or psychological, intellectual, moral or social development of the child or adolescent” and can be categorized into physical abuse, sexual abuse, emotional abuse, and neglect [2,3]. This review focuses specifically on physical abuse, also referred to as [2] “non-accidental injury”, “non-accidental trauma”, “child endangerment”, and “child maltreatment” in the literature. Regardless of the specific terminology used, deliberate physical harm to a child, unfortunately, remains a prevalent issue that demands the attention and management of pediatric healthcare providers. The objective of this review is to equip medical professionals with a structured framework for identifying and evaluating children presenting with traumatic injuries that may be secondary to abuse. 

## 2. Epidemiology

Non-accidental trauma (NAT) stands as a primary cause of pediatric traumatic injury and fatality worldwide [4,5]. According to the 2021 report by the United States Department of Health and Human Services (DHHS), substantiated cases of child abuse, encompassing various forms, were noted at a rate of 8.1 cases per 1000, with NAT accounting for a significant 16% of these cases [6]. It is suspected that the true incidence of NAT is substantially higher than cited in this report, as many cases go unreported or undiagnosed by medical professionals [7]. In fact, the Centers for Disease Control (CDC) estimates that one in seven children have experienced some form of abuse in the past year [8]. Similarly, the European Status Report on Preventing Child Maltreatment found in community surveys performed across Europe that the prevalence of physical abuse is 22.9% [9]. A study by Gilbert et al. in *The Lancet* revealed annual reported rates of NAT in households in high-income countries worldwide ranged from 3.7 to 16.3% [3]. Unreported cases of NAT carry a heavy burden, as victims face a staggering 30–50% risk of repetitive abuse and a 10% risk of death due to abuse [7].

The fatality rate stemming from NAT in the United States (US) is estimated at 1.05 children per 100,000, which translates to approximately one to two deaths per day [6,10] In Europe, fatality rates vary widely by country, ranging from 0.18 per 100,000 in Greece to 3.23 per 100,000 in Slovakia [9]. The youngest children bear the highest risk of mortality, with 66.2% of deaths occurring in children under three years old [4,6]. Children under 12 months of age are three times more likely to succumb to NAT compared to their older counterparts [11]. In the US, African-American children face a disproportionately higher risk of death due to NAT, with rates 2.9 times higher than White children and 3.8 times higher than Hispanic children [6]. Of note, children experiencing repeated episodes of abuse endure fatality rates 2.5 to 7 times higher than those with only one identified episode. This underscores the critical importance of early recognition and intervention by pediatric medical providers to prevent these tragic fatalities [4,11,12].

With all the psychosocial impacts of the global coronavirus pandemic in mind, several studies delved into the pandemic’s impact on NAT incidence and severity. During this period, the incidence of reported NAT appeared to decrease, but the fatality rate witnessed a distressing increase. The DHHS reported a 7.7% rise in fatality rates between their 2017 and 2021 reports, shedding light on the concerning trends in child abuse during times of heightened stress and vulnerability [6,13].

## 3. Demographics 

NAT disproportionately affects younger children, likely stemming from a multitude of reasons including their limited ability to communicate effectively. A quarter of all cases occur in children under one year of age [6,11,14]. There is an overall even distribution between male and female sexes [6,9,15]. In the US, African-American, American-Indian, and Alaskan Native children are all disproportionately affected at rates approximately double that of White children [6]. It is unclear, however, if these racial discrepancies are genuine reflections of risk or if they are influenced by providers’ biases in suspicion and the evaluation for abuse within these populations [11,16,17]. There is also a link to socioeconomic status as children hailing from households in the lowest income quartile are nearly four times more susceptible to NAT than those in the highest quartile [7]. Furthermore, NAT victims are more likely to reside in urban areas and be covered by Medicaid, in contrast to private health insurers [7,15]. Children with underlying psychological conditions, including anxiety, mood disorders, attention deficit disorder, disruptive disorders, and developmental disorders, face an increased risk of NAT [7,9]. In a review of over 1700 admissions for NAT, 60% of affected adolescents had a history of a psychological or neurological disorder [17].

**Figure 1:**
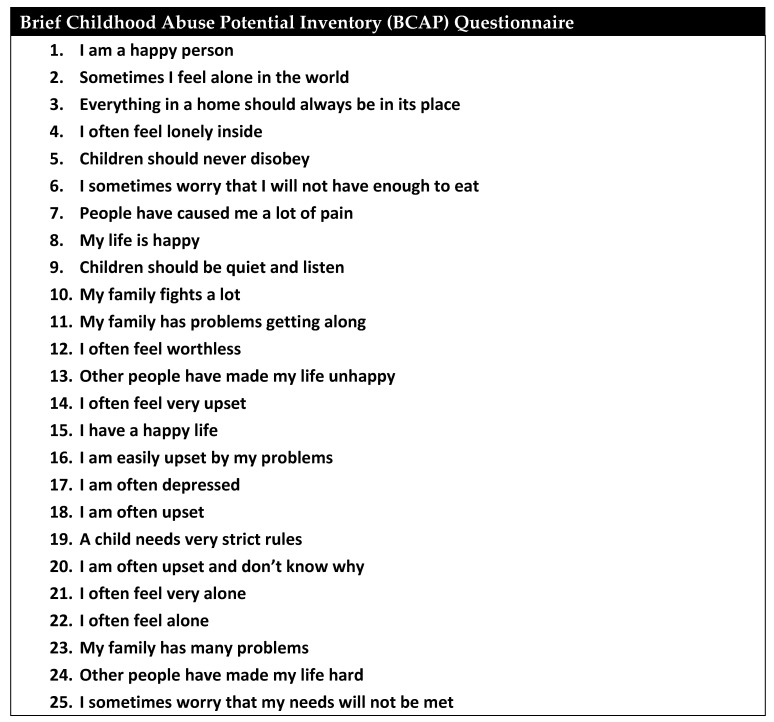
Brief Childhood Abuse Potential Inventory (BCAP).

Perpetrators of child abuse are overwhelmingly identified as the child’s parents or parental figures, with a breakdown of 38.0% attributed to mothers, 23.9% to fathers, and 20.0% to both parents [6]. When the data are broken down by abuse type, male parental figures are more associated with NAT, while female parental figures are more associated with neglect. Looking at male perpetrators in the National Child Abuse and Neglect Data System, 60% were biological fathers while 28% were social fathers (stepfathers, adoptive fathers, or boyfriends) [18]. Parental stress, whether stemming from psychological or financial pressures, is closely linked to an elevated risk of abusive behaviors [9,19]. Psychological disorders, such as a personal history of domestic, sexual, or childhood trauma, alcohol or substance use, and post-traumatic stress disorder (PTSD), are associated with higher rates of abusive behavior [9,20,21]. In addition to these risk factors, financial hardships, inadequate housing, food insecurity, criminal backgrounds, and reliance on public assistance further contribute to the occurrence of child abuse [6,19,22]. Efforts have been made to develop a scoring system aimed at identifying parents and other adults more prone to abusive behavior. Undesirable outcomes surrounding pregnancy, such as unwanted pregnancy, shorter interval pregnancies and perinatal illness, are all associated with increased likelihood of NAT [23]. The Childhood Abuse Potential Inventory (CAP), first published in 1986, comprises an extensive 160-question survey taken by suspected abusers and has been validated for its predictive value in recognizing the increased likelihood of abuse [24]. However, the questionnaire’s length limits its applicability. To address this concern, the Brief CAP (BCAP) was created as a condensed version, featuring 25 key elements, as illustrated in Figure 1 [24]. Nonetheless, even in its abbreviated form, the clinical utility of this questionnaire remains somewhat constrained and is primarily employed by psychologists rather than frontline clinicians. 

## 4. Patient Presentation

A vigilant clinician must consistently weigh the possibility of abuse when evaluating cases of pediatric trauma. Acquiring a comprehensive understanding of prevalent injuries, distinctive injury patterns, and historical elements linked to NAT can empower any healthcare provider tasked with the care of children to discern potential instances of abuse adeptly.

Numerous facets within the history of a pediatric trauma patient serve as crucial indicators for the pediatric provider to be vigilant for potential cases of NAT. In a review of 109 cases conducted at Wake Forest Baptist Health, the strongest historical predictor of underlying NAT was a discrepancy between the history provided and the identified injury [25]. Additional predictive factors encompass injuries misaligned with the child’s developmental stage, instances of trauma denial, conflicting narratives within the history, and delays in seeking medical care, all of which have been demonstrated as historically significant predictors of NAT [14,25]. 

## 5. Diagnosis and Evaluation 

**Figure 2:**
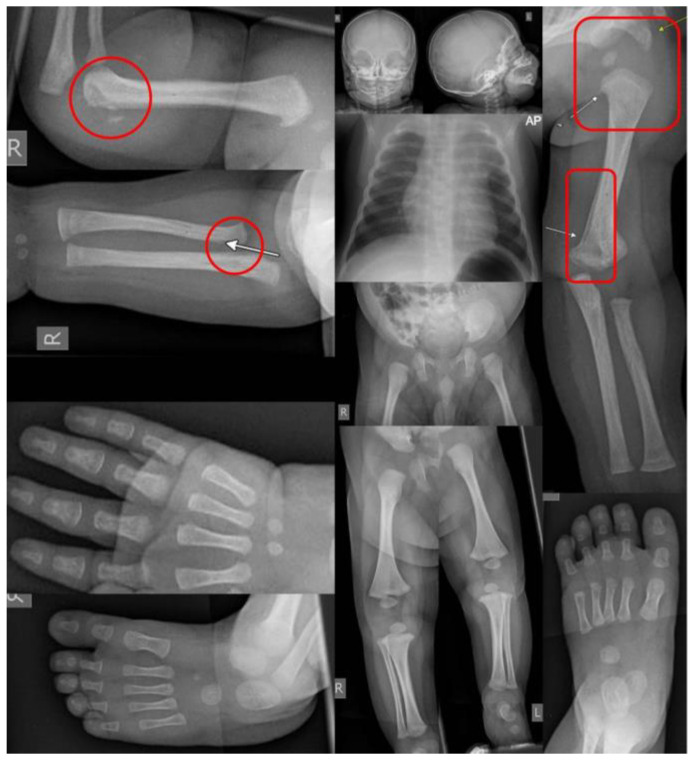
A skeletal survey of a four-month-old brought in with an inability to move the right arm, allegedly after the older sibling fell on top of him. The skeletal survey showed fresh corner fractures of the humerus and radius on the right (circle highlights) and signs of older fractures on the left (rectangular highlights), a combination that is pathognomonic for NAT.

A skeletal survey (SS) is a core component in the workup of patients suspected of NAT that involves the evaluation of the entire skeletal system for fractures (Figure 2). For children under two years old or those unable to communicate due to disability, the SS is universally recommended [11,14,26,27]. There is no standardized number of views required for a SS, and guidelines vary from 17 to 32 views, with an average of 20 views [26,28]. In general, orthogonal views of the entire axial and appendicular skeleton are recommended. Additionally, oblique views of the ribs are almost universally recommended [10,26,27,28,29,30]. Overall, this workup is considered low radiation with a dose of 0.2 mSV (millisievert) [28]. The use of SS has been quoted to identify occult fractures in up to a fourth of patients [31]. Additionally, many experts and organizations advocate for a repeat skeletal survey at an interval of 1–4 weeks in order to better identify occult fractures that become more apparent after callus formation begins [14,26,28]. This repeat SS typically contains fewer views than the primary SS with half of the radiation [28]. Unfortunately, despite the benefits of the repeat SS and expert recommendations for its use, compliance with this recommendation is reported to be low [28,31]. 

The use of bone scans for the evaluation of NAT is controversial due to the high dose of radiation. On average, a bone scan administers 3 mSV, which is approximately 10 times that of an SS [28]. Additionally, bone scans are often less specific than the SS as they can remain positive for years after fractures, and normal uptake by growth plates can create false positive findings [28]. Therefore, the use of a bone scan is only recommended in cases of a negative skeletal survey with a high index of suspicion [3,32].

To evaluate for abusive head trauma (AHT), the conclusive recommendation is for providers to perform a non-contrast head computed tomography (CT) scan. Most guidelines recommend systematic evaluation for AHT in all cases of suspected NAT, especially in children less than one year old, as many cases present without specific neurologic findings [14,26]. Magnetic resonance imaging (MRI) of the head is most consistently recommended in the case of positive CT findings for further specification [26]. To evaluate for spine injury, MRI is the recommended imaging modality of choice. Spine MRI is recommended in cases of positive findings on head CT and is to be performed concurrently with the MRI head. There are varying recommendations amongst guidelines if a full spine MRI is necessary or if the evaluation can be limited to the cervical spine [26]. 

Other imaging modalities are typically reserved based on the patient’s presentation. Imaging evaluation for abdominal trauma is most often recommended based on the clinical picture rather than systematically [26]. The decision to perform these studies can be advised based on laboratory studies or history and physical exam [26]. Recommended laboratory evaluation for intra-abdominal injury includes liver enzymes, pancreatic enzymes, urinalysis, and renal function evaluation [26,33]. Recommended cut-offs include transaminases greater than 80 IU/L [33,34]. The cut-off for lipase is less well defined, with a recommended cut-off between 50 and 100 IU/L [33]. Similar to head trauma, the PECARN (Pediatric Emergency Care Applied Research Network) abdominal injury rule is not an accurate clinical tool to utilize for patients with concern for abuse, since many exclusion criteria in this database would have excluded cases more likely to be due from NAT [35]. 

The imaging modality of choice for the evaluation of abdominal trauma is CT of the abdomen and pelvis with intravenous contrast [26,33]. Ultrasonography through the Focused Assessment with Sonography in Trauma (FAST) exam, while validated in the adult population, has been reported to have a sensitivity as low as 66–80% in the pediatric population [33]. Given these statistics, FAST is not a recommended imaging modality in the pediatric population [33]. MRI can be useful when utilized as follow-up imaging to clarify positive CT findings further but is not a recommended primary screening modality.

Radiation exposure is an important consideration in the pediatric population. Effective dose, often quantified in mSv, describes “the non-uniform exposure to ionizing radiation relative to whole body exposure” [32]. According to the National Academy of Sciences Bier VII Report, radiation doses of 100 mSv increase the lifetime risk of cancer by 1 in 100 [36]. While this risk is known to be higher in children given an increased radiosensitivity in growing tissues, the true risk is unknown. According to Bajaj and Offiah in their review, a dose of 2 mSv, the dose of an SS, suggests an additional lifetime risk of 0.032% [32]. Table 1 summarizes the effective radiation doses of the common imaging modalities utilized in the workup of NAT. Given the overall low rate of cancer compared to the high mortality of unrecognized NAT, imaging workup, when clinically indicated, should not be deferred because of concern for radiation. It is recommended, however, that every attempt is made to give a dose “as low as reasonably achievable” or “ALARA” [37]. 

### Interdisciplinary Approach

The optimal medical management for children affected by NAT necessitates a comprehensive and collaborative approach. In accordance with the guidelines from the American Pediatric Surgical Association, it is recommended that akin to any other trauma patients, those experiencing NAT should be admitted to a trauma surgical service. This is due to the established mechanisms in place to facilitate a thorough workup and seamless consultation with various subspecialty services that are crucial for comprehensive trauma care. This guideline is supported by numerous studies showing improved patient outcomes, fewer missed injuries, and more thorough follow-up when patients are admitted to a trauma surgery service [11]. Due to the variety of injury patterns associated with NAT, the care of these children requires coordination with diverse medical services, including but not limited to general pediatrics, neurosurgery, orthopedic surgery, and ophthalmology. Moreover, the involvement of non-clinical professionals such as social workers and child protective services is essential for coordinating care and protection for these patients once they are discharged from inpatient care. 

## 6. Injury Patterns

### 6.1. Soft Tissue Injury 

Bruising is the most prevalent injury seen in victims of NAT [42,43]. Other common soft tissue injuries include oral injuries, such as frenulum tears [44]. Frequently, these minor and clinically insignificant injuries serve as harbingers of abuse, and their identification by medical providers can serve as a pivotal opportunity for intervention before more serious injury occurs [14,19,43,45]. In one study, 27.5% of identified NAT cases had a previously documented sentinel bruise without a performed NAT workup, representing a missed opportunity for intervention before a more serious injury unfolded [44]. In a separate prospective study, 54% of infants presenting to their primary care provider (PCP) or an emergency department (ED) provider with unexplained bruises were ultimately found to be victims of NAT. Of these, 62% were subsequently diagnosed with occult injuries through further investigation [46]. 

**Figure 3:**
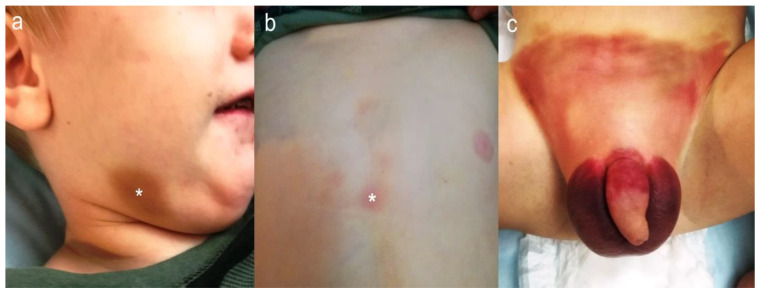
A two-year-old presented with right mandibular bruising suggestive of NAT (**a**). Upon physical examination, bruises were also found over the sternum (**b**) and the genital area (**c**). The asterisk (*) marks the most prominent bruises.

While bruising can be common in both accidental and non-accidental trauma alike, certain patterns of bruising should raise concerns for abuse. The developmental stage of the patient offers a significant clinical clue, as accidental bruising is a rare occurrence in infants who are not yet crawling or walking. [42,43,46]. Patterns of bruising can further heighten suspicion for a non-accidental source; for instance, bruising on bony prominences like the forehead, shins, and knees is more frequently linked to accidents, whereas bruising on soft tissues such as the ears, genitalia, buttocks, and cheeks is more frequently associated with NAT as shown in Figure 3 [14,42,43,45]. 

The TEN-4 FACES screening tool outlines the common NAT bruising locations in children aged less than five months [43,47]. In a cross-sectional study of 2161 patients, the presence of bruising in any of these identified locations was 95.6% sensitive and 87.1% specific for identifying NAT cases [43]. Additionally, the clustering of bruises, bruises in multiple stages of healing, or patterned bruising originating from a casual object, such as a hand, serves as a highly indicative marker of NAT [14,42,43]. 

In the case of multiple contusions, laboratory testing can rule out bleeding disorders as an underlying source. A complete blood count and coagulation panel are typically sufficient to begin this workup [26]. However, it is important to realize that bleeding disorders occur less frequently than NAT, and the workup for these disorders should not supersede the workup for NAT. 

### 6.2. Abusive Head Trauma 

Abusive head trauma encompasses all cranial or nervous system injuries secondary to non-accidental pediatric trauma [48]. Of all NAT injury types, AHT carries the highest fatality rate at approximately 25% [15,47,49]. This devastating form of abuse primarily operates through two key mechanisms: impact forces and inertial forces, which are often manifested as shaking [48,50]. The most prevalent intracranial pathologies resulting from AHT are complex subdural hemorrhage (SDH), hypoxic-ischemic injury, and skull fractures in conjunction with intracranial injury [47,48,51,52]. Additionally, retinal hemorrhages, typically resulting from shearing forces, are frequently linked to AHT. Retinal hemorrhage associated with AHT is typically bilateral and more diffuse when compared to accidental sources [47,52]. Traumatic retinoschisis, which is the accumulation of blood in the macula, is a highly specific finding for AHT [47]. Birth trauma and cardiopulmonary resuscitation are two mechanisms that have been used in litigation to suggest alternate sources for potential retinal hemorrhage as opposed to NAT; however, retinal hemorrhage due to birth trauma can be differentiated, as it is not multilayered and resolves two to four weeks after birth. Similarly, there have been no reported cases of retinal hemorrhage from cardiopulmonary resuscitation [11]. When assessing suspected cases of AHT, it is imperative to include a dilated fundoscopic examination performed by an experienced ophthalmologist, especially in children under one year of age [26,52].

Diagnosis of AHT can prove difficult as patients often present with nonspecific symptoms, such as fussiness or emesis, or they may be altogether asymptomatic [48,49]. A common historical explanation offered by parents is falls less than 5 feet, classified in the literature as “short falls”. However, there are few described cases of this injury pattern resulting in clinically significant intracranial injuries, and mortality from these injuries is incredibly rare at approximately one in two million. Therefore, when parents recount a history of short falls within the context of intracranial injury, it should instantly raise suspicion for AHT [47]. More specific findings include bulging fontanelles or evidence of retinal hemorrhage [48]. The PediBIRN-4 (Pediatric Brain Injury Research Network) is a clinical tool developed to aid in the identification of children who need screening for AHT. Patients presenting with one of four factors (apnea, bruising in TEN pattern, bilateral subdural hemorrhage, or complex skull fractures) are associated with a 96% sensitivity of associated AHT. Exclusion of complex skull fractures as a risk factor (known as the PediBIRN-3) has been shown to increase the accuracy of the tool but at a 3% sensitivity loss [53]. While the PECARN prediction rules are validated to pinpoint children who necessitate head imaging in the case of head trauma, this tool should not be employed for children with suspected AHT. As elucidated in a commentary by the first author of the original study validating the PECARN rules, this screening tool relies heavily on an accurate history, which cannot be reliably established in cases involving suspected abuse due to the inherent challenges in trustworthiness [54,55]. 

The primary imaging modality to evaluate for AHT is non-contrast CT [26]. Recently, however, there has been a growing trend towards the utilization of MRI [47,48]. CT offers several advantages, including greater accessibility, lower cost, and enhanced sensitivity in detecting skull fractures compared to MRI [48]. It also requires a shorter examination duration, thereby reducing the need for sedation [28]. However, these benefits must be thoughtfully balanced against the associated radiation risk, with radiation doses higher for diagnosing intraparenchymal injuries and its ability to assess potential cervical spine ligamentous injuries [48]. The general recommendation for evaluating AHT is to initiate the assessment with a CT scan, followed by an MRI at 2–5 days post-CT in cases of positive CT findings or for children with a negative CT but with high clinical suspicion for intracranial injury [26,28]. 

### 6.3. Skeletal Fractures

Fractures are a frequently encountered pathology in the pediatric population, with NAT making up only a small subset. In all pediatric admissions for fractures, NAT only represents 1% of cases [56]. Nonetheless, there exist distinctive fracture characteristics that more commonly align with NAT, thereby warranting heightened vigilance from medical providers. Age is the most significant predictive factor for identifying a non-accidental source [29,57,58]. In a study by Worlock et al., approximately 10% of fractures in children under 18 months are due to abuse, compared to 0.5% of fractures in children between 19 and 60 months [57]. Additionally, the presence of multiple fractures is five times more highly associated with abuse [57]. The simultaneous occurrence of other traumatic injuries, such as intracranial injury and burns, provides further predictive indicators [56]. 

In cases where patients present with a history of multiple fractures, it is imperative to exclude errors in bone metabolism as an underlying cause. Among the 20 guidelines addressing NAT workup, 7 recommend the evaluation of serum calcium, phosphorus, alkaline phosphatase, parathyroid hormone, and vitamin D levels. Some guidelines extend this workup to include the assessment of serum copper and ceruloplasmin as well [26]. 

### 6.4. Skull and Facial Fractures 

In a review of over 2 million pediatric hospitalizations, skull and facial fractures were identified as the most common fractures associated with NAT, seen in 27% of children [17]. Skull fractures, although equally linked to both AHT and accidental head trauma, exhibited distinct patterns in AHT cases, often characterized by increased fracture complexity [29,50]. Facial fractures are uncommon in the pediatric population due to the morphology of the pediatric face, characterized by a flatter midface and a larger cranium-to-face ratio [16]. However, within the realm of facial fractures, a review focusing on pediatric cases revealed that fractures resulting from abuse were more likely to occur in the mandible, with a specific emphasis on the mandibular condyle, rather than the midface [16]. 

### 6.5. Rib Fractures

Rib fractures are an uncommon injury in the pediatric population due to the increased plasticity of the pediatric chest wall. These fractures occur in only 2% of pediatric trauma patients, yet within the context of NAT, they are present in 10% of cases [10,17,30,39]. Despite their overall rarity, rib fractures have a strong association with NAT [39]. In a retrospective review by Barness et al., 65% of trauma patients presenting with rib fractures were victims of NAT [10]. In a separate review, rib fractures were shown to be more likely due to NAT than any other fracture type. Rib fractures due to NAT typically occur in younger children (<3 years) and are more numerous compared to accidental sources [10,29,30]. There is no clear association between the location of rib fractures and the injury source [10,29].

The diagnostic evaluation of rib fractures primarily involves radiography in the form of a skeletal survey (SS). Many experts advocate for the inclusion of oblique views in addition to anterior/posterior and lateral views in order to increase the sensitivity of radiographs [10,27,28,29,30]. Even with these additional views, rib fractures can still be missed on initial SS 16–66% of the time [28]. Follow-up skeletal survey increases the sensitivity [28]. For increased diagnostic sensitivity, CT of the chest can be used for the identification of rib fractures. However, this enhanced sensitivity comes at the cost of increased radiation exposure. A retrospective review comparing chest CT and SS for the evaluation of rib fractures noted a 17% miss rate by initial SS [39]. To mitigate radiation exposure, low-dose CT chest can be performed with only 0.5–0.7 mSV of radiation [39]. Currently, the decision to incorporate chest CT is contingent on the clinical suspicion of the healthcare provider and may include consideration of the likelihood of follow-up SS.

### 6.6. Appendicular Fractures

While fractures are common in children, suspicion should be raised for fracture patterns that are inconsistent with either a child’s developmental stage or with the provided injury history. Additional clues to a non-accidental source of fracture include concomitant burns and/or intracranial pathology, the presence of rib fractures, and multiple fractures [30]. The appendicular skeleton consists of the upper and lower extremities. This includes the shoulders and pelvis. The most common locations for extra-cranial fractures associated with NAT include the femur, humerus, tibia/fibula, and clavicle [7]. Beyond the fracture location, specific fracture patterns have a higher association with NAT. In the upper extremity, a retrospective review showed the uncommon transphyseal distal humerus fracture is thirteen times more likely to be from abuse compared to the more frequent supracondylar humerus fracture [59]. Femur fractures are common in both NAT and accidental trauma. Fifteen percent of femur fractures have been reported to be secondary to NAT [17]. Femur fractures in children under 24 months, transverse diaphyseal and metaphyseal femur fractures have higher associations with NAT [17,28,29,60]. While spiral fractures of the femur were originally thought to be highly correlated with NAT, the recent literature has shown no increased likelihood compared to transverse fractures [60,61].

### 6.7. Burns

**Figure 4:**
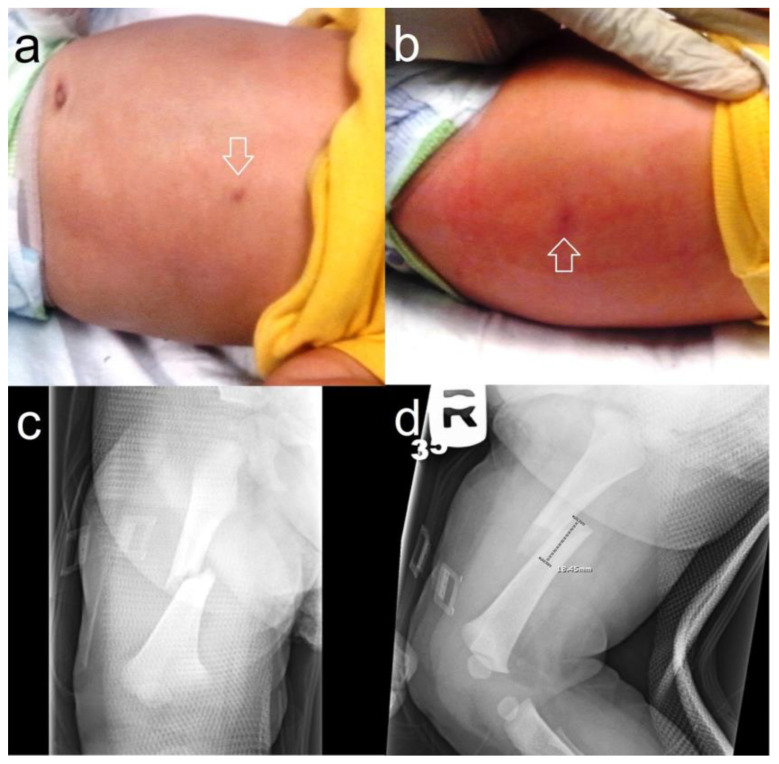
A nine-month-old was brought in for excessive irritability. Upon physical examination, there were two circular, healing burn marks on the abdomen (**a**) and back ((**b**), arrows) later proven to be from cigarette burns. A radiological workup and skeletal survey showed a right femoral fracture (**c**,**d**), but no other osseous anomalies.

Burn injuries impose a substantial burden of morbidity and mortality within the pediatric population, standing as the third most common cause of fatal injuries [62]. Up to a quarter of these cases result from NAT [62,63]. These non-accidental burns tend to exhibit deeper thickness compared to unintentional burns. The size and shape of the burn should be noted as they can provide clues into the source of the burn, such as cigarettes (Figure 4). Additionally, they are more often located on the posterior trunk, buttocks, and genitals and are more frequently bilateral [64]. The BuRN-Tool score is a screening tool to help providers identify which burn injuries require a higher index of suspicion for NAT. Patients presenting with three or more factors in this tool had a sensitivity of 82–88% and a specificity of 79–82% depending on the burn type. The associated factors are an age less than five, history, full thickness depth, uncommon body location, bilateral pattern, supervision concern, and the patient being known to social care [63].

### 6.8. Intra-Abdominal Injury

Intra-abdominal injury represents a rare but highly significant consequence of non-accidental trauma, affecting approximately 1% of children hospitalized for abuse but with mortality rates approaching 50% [65,66,67]. Similar to accidental sources, the most common abdominal injuries are injuries to solid organs [33]. However, compared to accidental sources of abdominal injury, abused children with abdominal injuries were three to eight times more likely to have hollow viscus involvement [33,65,68]. Additionally, pancreatic injury has a higher association with NAT [66,67,69]. Given the difference in injury patterns, providers should have a high index of suspicion if the identified injury does not correlate with the provided history. Similar to head trauma, short falls are one of the most commonly reported histories for patients presenting with intra-abdominal trauma. It is important for providers to know there is little documented evidence of hollow viscus injury in true cases of short falls and recognize the discordance of hollow viscus injuries and the reported history of a fall [66].

## 7. Outcomes

The profound repercussions of NAT extend well beyond the harrowing statistics on mortality that have already been discussed. Survivors of these traumatic events bear enduring burdens on their development, mental well-being, and physical health, with morbidity rates reaching levels of up to 50% [9,50].

NAT exerts a detrimental impact on cognitive development leading to lower cognitive scores, which can significantly hamper a child’s intellectual growth [70]. AHT is associated with 50–70% of neurological impairment including conditions such as cerebral palsy, intellectual disability, blindness, seizures, and learning disabilities [47,71]. These life-altering consequences of NAT underscore the dire need for early intervention and support for affected children.

Furthermore, there is a growing body of research shedding light on the long-term health outcomes for children who endure adverse childhood experiences (ACE), with NAT constituting a significant component of these experiences. ACE has been intricately linked to a host of adverse physical health outcomes, including a heightened risk of cardiac disease, malignancies, and obesity. NAT has been linked to reduced height compared to parental height and an increase in obesity [70]. Additionally, the toll on mental health is substantial, with elevated rates of substance abuse, depression, and suicide being associated with ACE [9,72]. NAT has been linked to increased rates of behavior problems and delinquency among survivors. This is hypothesized to stem from a state of hypervigilance acquired as a coping mechanism and a disproportionate response to cues perceived as threatening [70].

In the realm of mental health, victims of NAT are more likely to grapple with depression and PTSD [70]. Children who have been subjected to NAT have a 2.18-odds ratio of suicide attempts compared to children without this trauma [73].

## 8. Prevention

To make significant strides toward mitigating the morbidity and mortality stemming from NAT, a multifaceted approach that encompasses prevention, enhanced recognition of NAT cases, and ongoing advancements in treatment modalities is needed. Prevention strategies have proven to be particularly impactful in addressing NAT. These efforts revolve around two primary objectives: (1) raising awareness about NAT, with a specific emphasis on AHT, and (2) enhancing parental responses to child triggers [74]. The most widespread awareness program is the Period of PURPLE Crying [11,47,74]. This program is instrumental in educating parents about infant crying patterns and effective soothing techniques. Widely implemented in postpartum settings across the globe, it represents a pivotal step toward promoting parental awareness and responsiveness to reduce the incidence of NAT and associated harm to children.

## 9. Limitations

One limitation of this review is that it discusses the previously published literature and is not the first review article to be published on this topic. However, the authors felt that this review has value to front-line pediatric providers in its comprehensive evaluation of the current literature regarding the initial evaluation and workup of these challenging cases.

## 10. Conclusions

Non-accidental trauma is a widely prevalent and under-reported source of morbidity and mortality worldwide. Given the vulnerability of this population, increased awareness by any caretaker, including parents, teachers, and pediatric medical providers, is essential to decrease the incidence of NAT. Pediatric provider knowledge of the identifiable clinical signs, radiographic findings, and lab abnormalities linked to NAT is critical to identify and thoroughly work up any children suspected to be a victim. A high index of suspicion for any child presenting with an inconsistent or changing history in the setting of a traumatic injury is especially important. Finally, prevention is key to decreasing the significant impact that NAT has on children both during their childhood and throughout the rest of their lives.

## Figures and Tables

**Table 1:** Effective Radiation Dose of Imaging for NAT Workup.

Exposure	Average Effective Dose
Skeletal Survey (~17 images)	0.2 mSV [28,38]
Follow-Up SS (~10 images)	0.1 mSV [28,38]
Bone Scan	2.27–3.0 mSV [28,38]
CT Head	2–2.68 mSV [37,38,39]
CT Chest (low dose)	0.5–1.13 mSV [28,39]
CT Abdomen/Pelvis	5.06 mSV [37]
Annual Background Radiation Exposure in U.S.	3.1 mSV [40]
Flight From Frankfurt, Germany to New York, USA	0.050 mSV [41]

## Data Availability

Not applicable.

## Abbreviations

NAT	Non-accidental Trauma
DHHS	Department of Health and Human Services
US	United States
CDC	Centers for Disease Control and Prevention
CAP	Childhood Abuse Potential Inventory
CT	Computed Tomography
MRI	Magnetic Resonance Imaging
SS	Skeletal Survey
AHT	Abusive Head Trauma
PECARN	Pediatric Emergency Care Applied Research Network
FAST	Focused Assessment of Sonography in Trauma
mSV	Millisievert
PCP	Primary Care Provider
ED	Emergency Department
SDH	Subdural Hematoma
ACE	Adverse Childhood Experience
